# Evaluation of AI Performance in Spinal Radiographic Measurements Compared to Radiologists: A Study of Accuracy and Efficiency

**DOI:** 10.3390/jimaging11090310

**Published:** 2025-09-10

**Authors:** Francesco Pucciarelli, Guido Gentiloni Silveri, Marta Zerunian, Domenico De Santis, Michela Polici, Antonella Del Gaudio, Benedetta Masci, Tiziano Polidori, Giuseppe Tremamunno, Raffaello Persechino, Giuseppe Argento, Marco Francone, Andrea Laghi, Damiano Caruso

**Affiliations:** 1 Radiology Unit, Department of Surgical and Medical Sciences and Translational Medicine, Sapienza University of Rome, Sant’Andrea Hospital, 00189 Rome, Italy; guido.gentilonisilveri@uniroma1.it (G.G.S.); marta.zerunian@uniroma1.it (M.Z.); domenico.desantis@uniroma1.it (D.D.S.); michela.polici@uniroma1.it (M.P.); antonella.delgaudio@uniroma1.it (A.D.G.); benedetta.masci@uniroma1.it (B.M.); tiziano.polidori@uniroma1.it (T.P.); giuseppe.tremamunno@uniroma1.it (G.T.); raffaello.persechino@gmail.com (R.P.); giuseppe.argento@uniroma1.it (G.A.); marco.francone@uniroma1.it (M.F.); damiano.caruso@uniroma1.it (D.C.); 2 Department of Biomedical Sciences, Humanitas University, 20100 Rozzano, Italy; andrea.laghi@hunimed.eu; 3 Department of Diagnostic Imaging, IRCCS Humanitas, Research Hospital, 20100 Rozzano, Italy

**Keywords:** artificial intelligence, weight-bearing radiography, Cobb angle, spinal parameters, workflow efficiency

## Abstract

This study aimed to evaluate the reliability of an AI-based software tool in measuring spinal parameters—Cobb angle, thoracic kyphosis, lumbar lordosis, and pelvic obliquity—compared to manual measurements by radiologists and to assess potential time savings. In this retrospective monocentric study, 56 patients who underwent full-spine weight-bearing X-rays were analyzed. Measurements were independently performed by an experienced radiologist, a radiology resident, and the AI software. A consensus between two senior experts established the ground truth. Lin’s Concordance Correlation Coefficient (CCC), mean absolute error (MAE), ICC, and paired *t*-tests were used for statistical analysis. The AI software showed excellent agreement with human readers (CCC > 0.9) and demonstrated lower MAE than the resident in Cobb angle and lumbar lordosis measurements but slightly underperformed in thoracic kyphosis and pelvic obliquity. Importantly, the AI significantly reduced analysis time compared to both the experienced radiologist and the resident (*p* < 0.001). These findings suggest that the AI tool offers a reliable and time-efficient alternative to manual spinal measurements and may enhance accuracy for less experienced radiologists.

## 1. Introduction

Back pain is among the most frequent reasons for visiting a general practitioner or physiotherapist. Although magnetic resonance (MR) conducted in a supine position represents the gold standard for the detection of causes of back pain such as disk degeneration, Modic changes, and facet joint degeneration and offers many advantages in terms of soft tissue resolution and the absence of the use of ionizing radiation, it cannot accurately evaluate scoliosis, degree of thoracic kyphosis, lumbar lordosis, and pelvic obliquity. It is essential that spine X-rays are performed under weight-bearing conditions for the assessment of these parameters [1,2].

Manual measurements have several significant limitations. First, they are time-consuming, particularly when multiple measurements are required. Furthermore, their accuracy depends on the skills and experience of the operator, which can result in both inter-observer and intra-observer variability [3,4]. In this context, technological advancements have led to the increasing application of artificial intelligence (AI) in musculoskeletal (MSK) imaging [5,6,7,8]. AI is being increasingly adopted as a support tool to enhance the efficiency and accuracy of radiological assessments, providing fast, reliable, and reproducible measurements while minimizing errors caused by distraction and fatigue [9,10,11]. 

Recent advancements in artificial intelligence (AI), the capability of computational systems to perform tasks typically associated with human intelligence, such as learning, reasoning, perception, and decision-making, have opened up new frontiers in medical imaging, particularly in musculoskeletal radiology. AI-based tools promise to increase efficiency, reduce variability, and support less experienced radiologists in complex measurements. In this context, several algorithms have already been proposed for tasks such as automated Cobb angle quantification, hip morphology assessment, and spinopelvic parameter estimation. These models, often based on convolutional neural networks or U-Net–like segmentation pipelines, have shown promising accuracy and reproducibility across different clinical scenarios. However, growing evidence suggests that the integration of AI into clinical workflows does not always yield uniform benefits. A recent large-scale study by Harvard Medical School and collaborators [12] revealed that AI assistance may improve or impair diagnostic performance depending on the individual radiologist’s characteristics, highlighting the need for tailored AI implementation. In this context, evaluating the reliability, accuracy, and practical utility of AI tools in real-world radiological tasks is essential. The integration of AI into radiology education is gaining momentum, with growing interest among faculty, residents, and medical students. A recent survey by Hoyer et al. [13] highlighted the perceived value of AI-powered platforms for enhancing learning through interactive case-based training, automated feedback, and simulated reporting. While enthusiasm for AI in education is high, the study also revealed the need for user-centered design, transparency, and alignment with learners’ expectations. These findings underscore the importance of carefully structured AI integration—not only in clinical workflows but also in radiological training and assessment.

The primary aim of this study is to evaluate the reliability of an AI-based software tool, BoneMetrics (version 2.3.1, Gleamer, Paris, France), chosen because it is currently implemented in our institution and routinely used in clinical practice, in measurements of different spine parameters and to compare its accuracy to radiologists. Our secondary aim was to quantify time savings achieved using the automated software compared to traditional manual methods. The novelty of this is that, unlike prior works, we compared AI not only with an expert radiologist but also with a resident, thus exploring its role in supporting less experienced readers. Our main contributions are (i) validation of Bone Metrics in spine imaging, (ii) benchmarking against readers with different expertise, and (iii) demonstration of workflow efficiency gains. The study followed a simple retrospective framework with expert consensus as the reference standard and standard statistical analysis.

## 2. Materials and Methods

### 2.1. Patient Population

We conducted a retrospective monocentric study at Sant’Andrea Hospital, Sapienza University of Rome, and enrolled patients who underwent whole-spine weight-bearing X-rays from January 2023 to October 2024. Patients who refused to provide informed consent and images with severe artifacts were excluded from the study. Examinations were performed for different clinical indications: in pediatric patients, they were performed mainly for suspected scoliosis, while in adult patients, they were performed for degenerative spine diseases and suspected spinal instability. This study was approved by the IRB (approval code CE 6597/2021), and informed consent was obtained from all participants.

### 2.2. Image Acquisition

**Figure 1:**
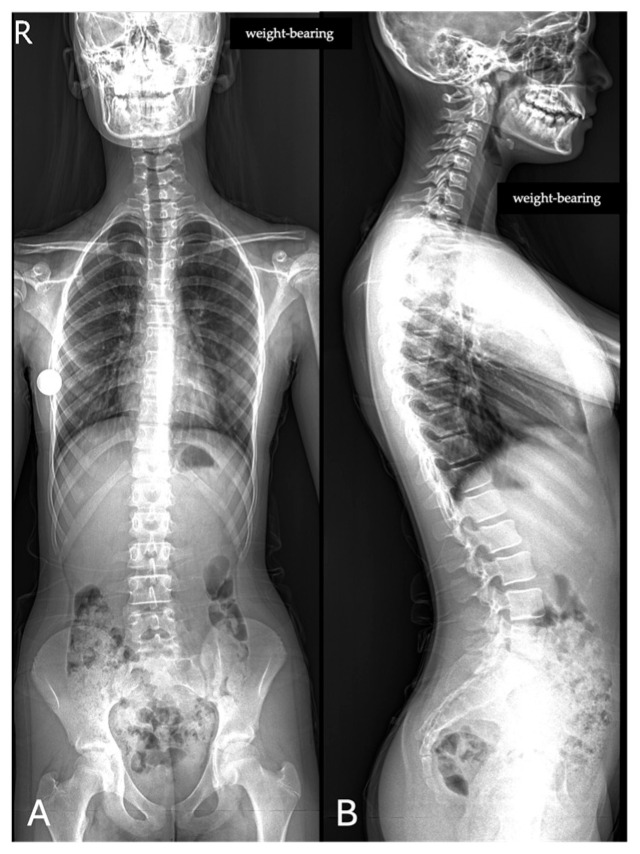
Full-spine radiographs were acquired in standing position using the same dedicated scanner (Bloomix 120 ED-R). (**A**) Anteroposterior (AP) view; (**B**) lateral view. For each patient, AP and lateral projections were obtained from the base of the skull to the last sacral segment, including the hip bones and femoral heads. The scanner enabled the acquisition of the entire spine in a single shot, without the need for image stitching.

All images were acquired using the same digital radiography scanner (Bloomix 120 ED-R, Trade Art Manifacturing, Via della Pisana 1353, 00163 Rome, Italy) with patients in a standing weight-bearing position, in order to reproduce physiological load conditions on the spine. For each patient, both anteroposterior (AP) and lateral projections were obtained, covering the entire spine from the second cervical vertebra (C2) to the last sacral vertebral body (S5), including the pelvic bones and both femoral heads to allow evaluation of spinopelvic parameters. The dedicated scanner used in this study enables the acquisition of the entire spine in a single exposure, without the need for image stitching, thus reducing potential misalignment artifacts and ensuring greater measurement accuracy. Standardized positioning protocols were adopted to minimize patient rotation and to maintain consistent image quality across all examinations. All acquisitions were performed by experienced radiographers according to the department’s routine protocol for whole-spine evaluation. Exposure parameters, such as tube voltage and current, were adjusted based on patient size and age to optimize image quality while maintaining the radiation dose as low as reasonably achievable (ALARA principle) (Figure 1).

### 2.3. Image Analysis

Image analysis was performed independently by a radiologist with 25 years of experience in musculoskeletal (MSK) radiology (GA) and by a radiology resident (GG) with 3 years of experience. Subsequently, the images were also analyzed by the AI software. The AI-based software, BoneMetrics (version 2.3.1, Gleamer, Paris, France), is a CE-certified AI-based software tool designed to automatically perform musculoskeletal measurements on conventional radiographs and EOS acquisitions. The system integrates several convolutional neural network models for the detection and localization of anatomical landmarks, from which quantitative parameters are subsequently derived. For each landmark, the algorithm provides a confidence score ranging from 0 to 100; only points exceeding a threshold of 50 are included in the final measurement process. The software architecture combines different approaches, including a top–down framework based on Detectron2, a lightweight high-resolution network (LiteHRNet), and a bottom–up strategy. This multi-architecture design enhances the adaptability and robustness of the model across diverse radiographic datasets. The training dataset comprised more than 5000 radiographs collected from over 20 European centers, including cases both with and without orthopedic implants. Annotation was performed by radiographers and radiologists with specific training and subsequently reviewed by an experienced musculoskeletal radiologist with over 14 years of expertise to ensure data quality. Importantly, none of the radiographs or patients included in the current study were used in the training phase of the software [14].

The radiologists were blinded to the measurements obtained by the AI system. The following measurements were performed: Cobb angle (defined as the angle formed by lines drawn parallel to the endplates of the two vertebrae that exhibit the greatest inclination relative to the horizontal axis) and pelvic obliquity (determined as the distance between the pelvic coronal reference line and a horizontal line aligned parallel to the floor, measured on the AP view); thoracic kyphosis (measured as the angle formed by two lines passing through the superior endplate of T1 and the inferior endplate of T12); and lumbar lordosis (measured as the angle formed by two lines passing through the superior endplate of L1 and the inferior endplate of L5), calculated on the lateral view.

All measurements were performed directly with the PACS system (GE Healthcare, Waukesha, WI, USA) using the standard built-in tools for measuring angles and distances, without the use of dedicated post-processing workstations or advanced image analysis software [12,13].

For validation of the measurements obtained by both radiologists and the AI tool, an orthopedic surgeon with 35 years of experience in spine surgery (NM) and another radiologist with 15 years of experience in musculoskeletal (MSK) radiology (RP) were considered as the reference standard (ground truth).

In addition, the time required to perform the measurements was recorded for both human readers and the AI software, in order to assess the potential impact on workflow efficiency.

### 2.4. Statistical Analysis

Statistical analyses were performed using MedCalc (MedCalc Software, version15, Ostend, Belgium). Data were reported as mean ± standard deviation (SD) or as absolute differences, depending on the analysis type. Paired *t*-tests were used to compare measurements between the radiologists and AI. Separate analyses were conducted for each parameter, including Cobb angle, thoracic kyphosis, lumbar lordosis, and pelvic obliquity.

The agreement between the experienced radiologist, radiology resident, and AI tool was assessed using Lin’s Concordance Correlation Coefficient (CCC). CCC values greater than 0.90 were interpreted as showing excellent agreement. In addition, inter- and intra-observer reliability were assessed by calculating the Intraclass Correlation Coefficient (ICC, two-way random effects, absolute agreement). ICC values above 0.90 were considered indicative of excellent reliability.

The accuracy of the resident and AI was evaluated by calculating the Mean Absolute Error (MAE) relative to the expert’s measurements, which were used as the reference standard. 

The statistical significance threshold was set at *p* < 0.05.

## 3. Results

### 3.1. Patient Population

**Figure 2:**
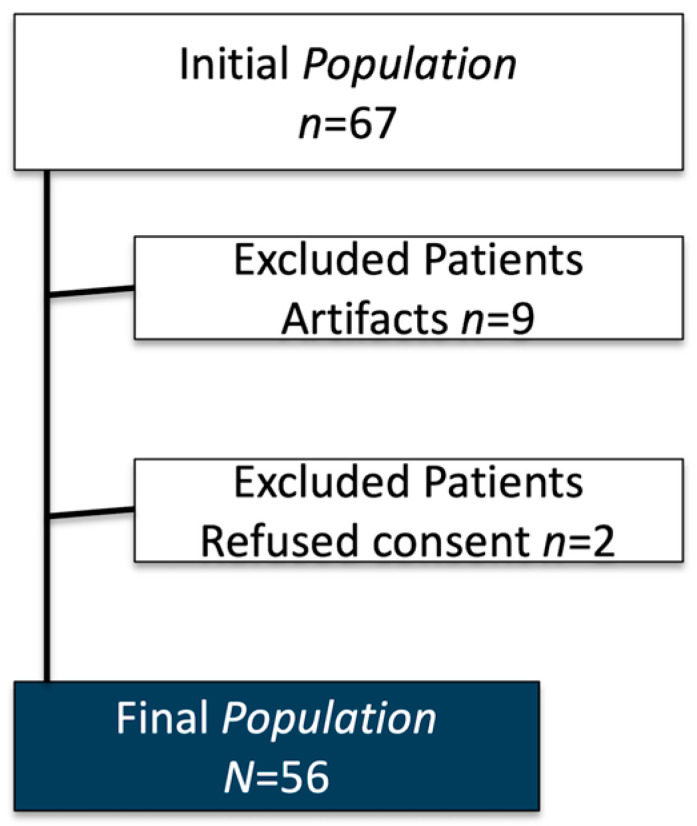
Patient enrolment flow-chart.

From an initial population of 67 patients, a total of 56 were included in the study (21 males, 35 females). The participants had a mean age of 33.65 ± 24.51 years (range 7–74 years). Among the included subjects, 23 were underage patients. Images with severe artifacts (*n* = 9) and patients who refused to provide informed consent (*n* = 2) were excluded from the analysis. Pathological findings were detected in 49/56 patients (87.5%). Specifically, scoliosis was found in 16/56 patients (28.6%), spondylolisthesis was found in 4/56 patients (7.1%), and a vertebral collapse was identified in 1/56 patient (1.8%) (Figure 2).

### 3.2. Image Acquisition

A total of 122 projections were acquired. In six patients, additional projections were required due to incorrect positioning, which initially prevented accurate measurements. For full-spine acquisitions, correct shoulder positioning in the lateral (LL) view was essential to avoid superimposition of the cervico-dorsal junction, a key reference for evaluating vertical axis alignment. In the anteroposterior (AP) view, the mean tube voltage was 82 kV (range 80–90 kV), with a tube current of 200 mA, an exposure of 64 mAs, and a time of 320 ms. In the lateral view, the mean tube voltage was 84 kV (range 80–90 kV), with a tube current of 200 mA, an exposure of 80 mAs, and the same acquisition time of 320 ms.

### 3.3. Image Analysis

No significant differences were found in any of the measurements between AI and the experienced radiologist or the radiology resident (all *p* > 0.05). Likewise, no differences emerged between the two human readers (all *p* > 0.05). Finally, no statistically significant differences were observed between pediatric and adult patient groups (*p* > 0.05) (Table 1).

**Figure 3:**
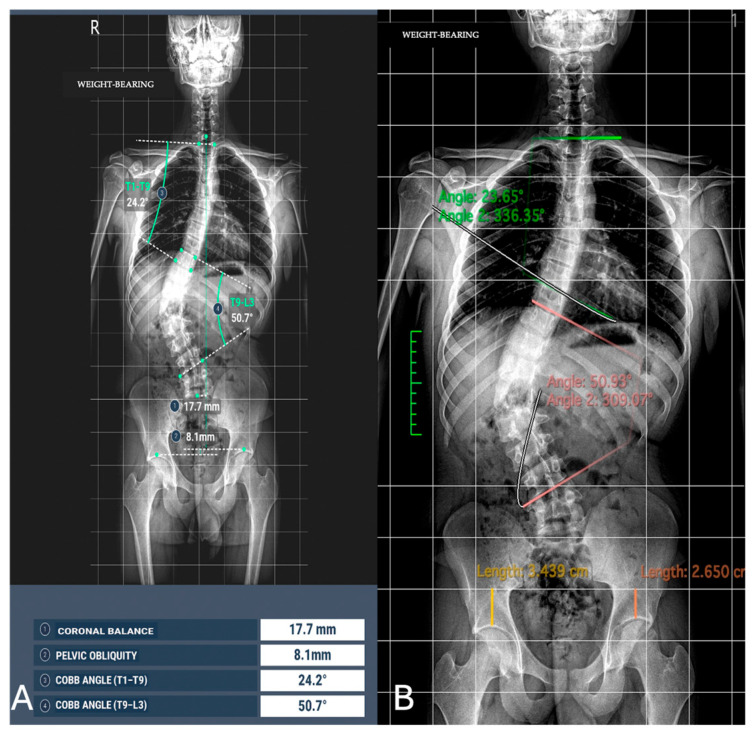
Image analysis of Cobb angle and pelvic obliquity in the same patients. (**A**) Automated measurements; (**B**) radiologist measurements.

**Figure 4:**
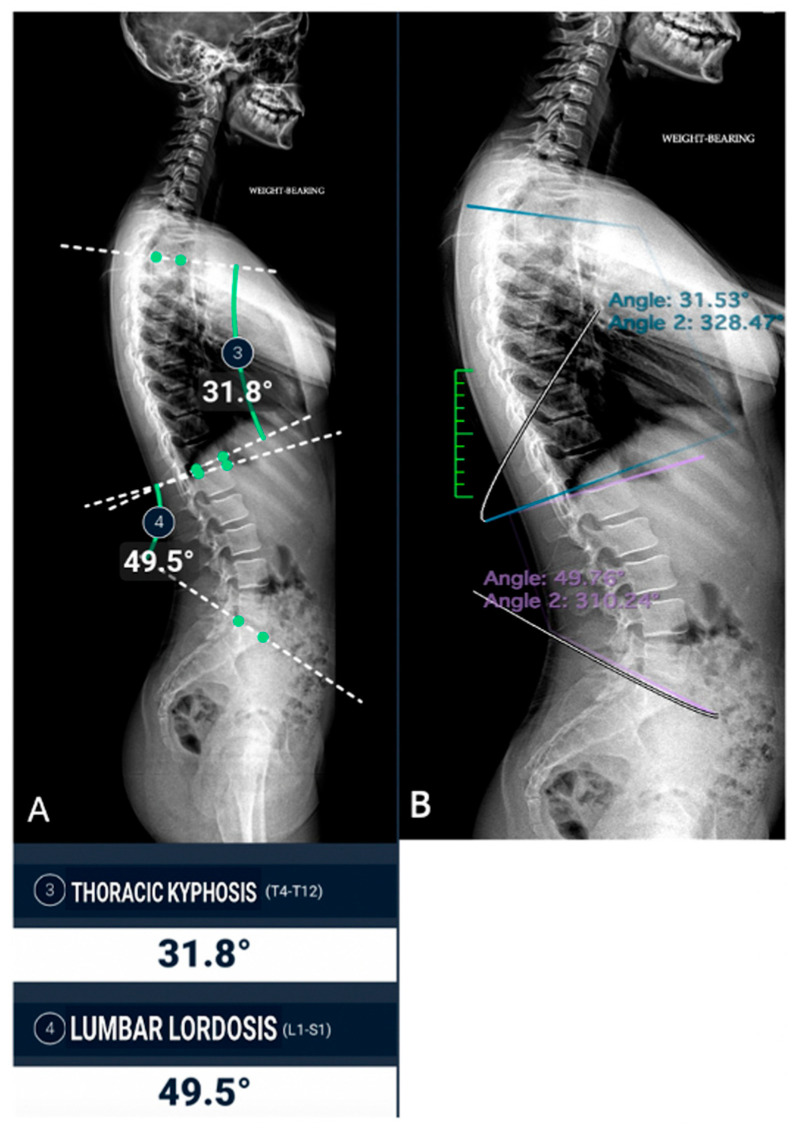
Sagittal spine image analysis in the same patient. (**A**) Automated measurements of thoracic kyphosis (T4–T12) and lumbar lordosis (L1–S1); (**B**) manual measurements performed by a radiologist.

The concordance between measurements by the experienced radiologist, radiology resident, and AI demonstrated a high level of agreement across all parameters, with CCC values exceeding 0.9 in most cases (Figure 3 and Figure 4).

For the Cobb angle, the AI achieved a lower MAE (0.577) compared to the resident (0.632), indicating slightly greater accuracy. For thoracic kyphosis, the resident exhibited a lower MAE (2.375) compared to the AI (2.464), suggesting better agreement with the expert in this measurement. In the case of lumbar lordosis, the AI demonstrated superior accuracy with a lower MAE (1.395) compared to the resident (1.902). Conversely, for pelvic obliquity, the resident achieved a lower MAE (0.296) compared to the AI (0.475) (Table 2). The inter-observer agreement between the experienced radiologist and the resident was excellent for all parameters (ICC range 0.905–0.996). When including the AI software as a third reader, the overall concordance remained excellent (ICC range 0.931–0.996). In addition, intra-observer reproducibility was also excellent across all parameters (ICC > 0.90).

**Figure 5:**
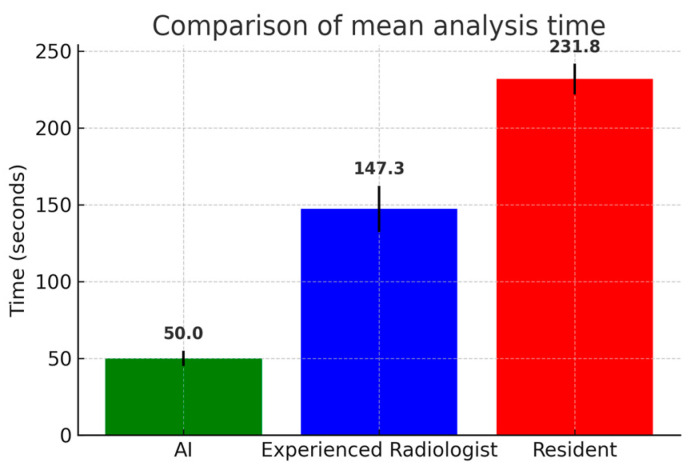
Comparison of mean analysis time (seconds) among artificial intelligence (AI) software, the experienced radiologist, and the radiology resident. AI software required significantly shorter analysis times compared to both human readers, while the experienced radiologist outperformed the resident. Error bars represent standard deviations.

Analysis time was significantly shorter for AI vs. the experienced radiologist (50.00 ± 0.00 s vs. 147.34 ± 18.78 s, *p* < 0.001) and for AI vs. the radiology resident (50.00 ± 0.00 s vs. 231.78 ± 20.15, *p* < 0.001). Analysis was significantly faster for the experienced radiologist vs. the radiology resident (147.34 ± 18.78 s vs. 231.78 ± 20.15, *p* = 0.027) (Table 3) (Figure 5).

## 4. Discussion

Our study demonstrated that the AI tool BoneMetrics (version2.3.1, Gleamer, Paris, France) has the same accuracy as an experienced radiologist and radiology resident in measuring Cobb angle, pelvic obliquity, thoracic kyphosis, and lumbar lordosis on a weight bearing spine X-ray with a significant analysis time reduction. Furthermore, if not significant, a *p*-value (0.062) for Cobb angle measurements between the AI tool and radiology resident suggest that this tool could help non-experienced radiologists with this measurement. The AI system performed better than the resident in some parameters, such as the Cobb angle and Lumbar Lordosis, while the resident showed better performance in others, such as Thoracic Kyphosis and Pelvic Obliquity. These results suggest that leveraging both AI and human expertise could optimize accuracy and reliability in radiological assessments.

Up to now, this study is the first to test this specific AI tool on the spine. Lassalle et al. [14,15] in their multicentric study demonstrated a comparable performance of this AI tool on weight-bearing forefoot and lateral foot radiographs, with relevant time savings. Similarly to our work, their study was retrospective and based on senior radiologists’ annotations as the ground truth, with only slightly more patients included. Unlike their study, however, our results highlight the additional value of this tool in supporting less experienced radiologists. Suri A. et colleagues [16], in their retrospective study, with experienced radiologists as the ground truth, presented a deep learning algorithm (SpineTK) trained with a fivefold cross-validation scheme and hyperparameter tuning to avoid overfitting, which is also available as open-source code. Their model demonstrated a strong agreement with manually annotated Cobb angle measurements performed by experienced radiologists and was statistically invariant across several clinical characteristics (hardware presence, sex, BMI, scoliosis severity, age, and image type). Moreover, they reported >90% accuracy in detecting scoliosis across all severity grades, and the algorithm proved robust in challenging clinical conditions such as severe scoliosis, different imaging positions, and the presence of extensive spinal hardware. Unlike previous studies such as Li et al. [17], where an AI algorithm (a multi-stage CNN-based pipeline consisting of spine region detection, vertebral segmentation with a U-Net-like architecture, centerline extraction, and automatic identification of the most tilted vertebrae) was used to automate Cobb angle measurements exclusively on the coronal plane, our study evaluated spinal alignment on both the sagittal and coronal planes. In the work by Li et al., the automated method showed a mean absolute deviation of 4.17° from expert readers, which was even lower than the average intra-reader variability (5.16°). However, their analysis was limited to scoliosis assessment alone. In contrast, our approach incorporates both coronal Cobb angle (scoliosis) and sagittal curvature metrics (e.g., thoracic kyphosis and lumbar lordosis), offering a more comprehensive characterization of spinal deformities [17].

Boel F. and colleagues [18], in their single-center study, investigated the agreement and reliability of manual and automated morphological measurements on AP pelvic radiographs for different measurements prospectively conducted in a population of 30 patients, and the mean of measurements performed by experienced orthopedic surgeons was used as the reference standard. Their presented algorithm (an automated pipeline based on BoneFinder® software, v.1.3.4 for landmark detection and a Python-based framework for radiographic measurement derivation) performed equally well compared to the current best practice of manual measurement by trained readers. By automatically annotating 80 anatomical landmarks on AP pelvic radiographs and subsequently deriving hip morphology indices, the system demonstrated both reliability and efficiency, while significantly reducing the time required for radiological measurements. 

The performance of an algorithm developed with deep-learning and computer vision techniques, using a modified U-Net architecture with a ResNet34 backbone trained on more than 2900 pelvic radiographs for measurement of the lateral center edge angle of Wiberg (LCEA) and Acetabular index angle (AIA) when reading pelvic radiographs, was investigated by Jensen J. et al. [19]. Like our work, their study was retrospective, with only slightly more patients included (71); the lack of a ground truth against which the algorithm and human measurements could be compared was also a limitation for them. The manual identification of the same landmarks performed by five human readers was subject to variance, and the level of agreement between the algorithm and human readers was consequently poor, although a tendency was revealed for the senior orthopedic surgeon to agree the most with the algorithm, particularly for LCEA right measurements. Our results, compared to those obtained in these other studies, have shown the same performance for AI tools and experienced radiologists. The strength of our study is in the potential role of this AI tool to help non-expert MSK radiologists. 

Our results are consistent with recent evidence by Harake et al. [20], who developed SpinePose, which employs three parallel CNNs: two top–down region-based models for L1 and S1 and a bottom–up encoder–decoder model for the remaining key points. This architecture enables precise localization of anatomical landmarks by combining region-specific detection with direct key point prediction on the native image. In their study, the model was trained on 761 lateral radiographs and demonstrated mean absolute errors (MAEs) ranging from 3° to 5° across parameters such as pelvic incidence, pelvic tilt, sacral slope, and lumbar lordosis. Furthermore, coefficient of determination (R^2^) values ranged between 0.85 and 0.92, confirming high consistency with manual annotations performed by experienced spine surgeons and neuroradiologists. These findings reinforce the idea that AI is not only a valuable tool for radiologists but can also support a broader spectrum of specialists, including orthopedic surgeons and physiatrists, particularly in the context of preoperative planning and spinal alignment evaluation. 

Beyond quantitative performance metrics, recent evidence also highlights a growing positive perception of AI among radiologists. For example, in the prospective study by Hoppe et al. [21], the integration of AI tools not only improved diagnostic workflow but also enhanced radiologists’ confidence and acceptance of AI in clinical practice. Participants reported increased trust in the technology and acknowledged its utility as a support tool, especially in high-pressure settings. Similarly, in our study, the AI tool was not only accurate and time-efficient but also well-received by radiologists during implementation, suggesting that the integration of AI into spinal imaging can be perceived as a helpful and reliable adjunct rather than a threat to professional expertise. These findings support a broader acceptance of AI in radiology, where clinical utility, efficiency, and user perception are all essential in real-world adoption. 

While our study, along with others, demonstrates the quantitative benefits of AI tools in terms of accuracy and efficiency, the integration of artificial intelligence into clinical radiology workflows also requires careful consideration of practical challenges, as highlighted by the recent literature. For instance, in their study, Korfiatis et al. [22] emphasize issues such as data standardization, integration into existing PACS/RIS systems, regulatory compliance, and the importance of clinician trust and training. These considerations are crucial in ensuring the real-world adoption and scalability of AI technologies.

The retrospective nature of the study and the small number of participants could be considered to represent the first limitation of the study. Furthermore, the monocentric nature could be considered a second potential limitation. A third limitation to the current study is the lack of an objective ground truth against which the algorithm and human measurements can be compared. Establishing a ground truth is, however, a common challenge in radiographic measurements. A fourth limitation is that the study was restricted to BoneMetrics, as it is the only AI tool currently implemented in our institution.

## 5. Conclusions

In conclusion, BoneMetrics demonstrated comparable accuracy to both an experienced radiologist and a radiology resident in measuring Cobb angle, pelvic obliquity, thoracic kyphosis, and lumbar lordosis on weight-bearing spine radiographs, while significantly reducing the analysis time. These results suggest that AI-based tools can provide a reliable and time-efficient alternative for routine clinical practice. Importantly, the use of BoneMetrics may be particularly valuable in supporting less experienced radiologists, ensuring consistency and reducing inter-observer variability. Future studies with larger and more diverse patient populations will be essential to further validate these findings and to explore the broader integration of AI into musculoskeletal imaging workflows.

## Figures and Tables

**Table 1:** Mean values (±standard deviation) for Cobb angle, pelvic obliquity, lumbar lordosis, and thoracic kyphosis as evaluated by the radiologist, the resident, and artificial intelligence software.

	Cobb Angle (°)	*p* Value	Pelvic Obliquity (mm)	*p* Value	Thoracic Kyphosis (°)	*p* Value	Lumbar Lordosis (°)	*p* Value
AI vs. Experienced Radiologist	12.75 ± 11.46	0.110	2.49 ± 1.08	0.572	38.42 ± 11.47	0.336	58.25 ± 11.74	0.282
AI vs. Radiology Resident	12.75 ± 11.46	0.062	2.49 ± 1.08	0.336	38.42 ± 11.47	0.062	58.25 ± 11.74	0.135
Experienced Radiologist vs. Radiology Resident	12.57 ± 11.27	0.356	2.53 ± 1.06	0.562	38.04 ± 11.81	0.127	57.83 ± 11.40	0.265

**Table 2:** Comparison of mean absolute error (MAE) and Concordance Correlation Coefficient (CCC) between the expert, the resident, and artificial intelligence (AI) software for the evaluation of Cobb angle, thoracic kyphosis, lumbar lordosis, and pelvic obliquity.

	MAE (Resident)	MAE (AI)	CCC (Expert–Trainee)	CCC (Expert–AI)	CCC (Trainee–AI)
COBB ANGLE	0.632	0.577	0.996	0.997	0.994
THORACIC KYPHOSIS	2.375	2.464	0.904	0.913	0.989
LUMBAR LORDOSIS	1.902	1.395	0.909	0.984	0.898
PELVIC OBLIQUITY	0.296	0.475	0.994	0.988	0.988

**Table 3:** Comparison of analysis time (seconds) between the artificial intelligence (AI) software, experienced radiologist, and radiology resident.

	Analysis Time (Seconds)		*p*-Value
AI vs. Experienced Radiologist	50.00 ± 0.00	147.34 ± 18.78	*p* < 0.001
AI vs. Radiology Resident	50.00 ± 0.00	231.78 ± 20.15	*p* < 0.001
Experienced Radiologist vs. Radiology Resident	147.34 ± 18.78	231.78 ± 20.15	*p* = 0.027

## Data Availability

The original contributions presented in this study are included in the article. Further inquiries can be directed to the corresponding author.

## Abbreviations

The following abbreviations are used in this manuscript:

AI	Artificial Intelligence
MSK	Musculoskeletal
MR	Magnetic Resonance
AP	Anteroposterior
LL	Latero-lateral (Lateral View)
CCC	Concordance Correlation Coefficient
MAE	Mean Absolute Error
IRB	Institutional Review Board
